# Supplementary material for: Synchrotron “virtual archaeozoology” reveals how Ancient Egyptians prepared a decaying crocodile cadaver for mummification
Source: PLoS One. 2020 Feb 21;15(2):e0229140. doi: 10.1371/journal.pone.0229140 (PMC7034907; doi:10.1371/journal.pone.0229140)
Supplement: Attachment — Submitted filename: PONE-D-19-24542 review.pdf [file pone.0229140.s002.pdf]

PONE-D-19-24542

Referee: Jana Jones, PhD. Macquarie University, Sydney Australia.

Synchrotron "virtual archaeozoology" reveals how Ancient Egyptians prepared a decaying crocodile cadaver for mummification

This paper presents an examination of a mummified Nile crocodile (*Crocodylus niloticus*) in the collection of the Musée de Confluences (Lyon, France) utilising the unique properties of the synchrotron rather than traditional light sources. The authors claim that the animal must have been in an advanced state of decomposition when mummified, because the internal organs, muscles and the majority of the bones had been removed prior to treatment. Their main deduction is that this is evidence of a mummification protocol different to that attested to in texts and archaeological sources, showing the diversity of techniques practised by the ancient Egyptians. They propose that this preparation protocol was applied to halt further putrefaction. Furthermore, the authors have duplicated the preparatory process on a recently deceased Caiman crocodile (*Caiman crocodilus*).

The authors have conducted a review of the early literature on the animal mummy collection in the Musée de Confluences. This collection was assembled mainly by Lortet and Maspero in the late 19th and early 20th centuries and the specimens were subsequently unwrapped and cleaned. They have cited the work of modern imaging studies that have shown that votive mummies were often an assemblage of disparate body parts but have found no evidence of treatment similar to that of the specimen under study.

There is no provenance recorded in the early literature, but the authors suggest Kom Ombo and Esna as possible sources. It is unfortunate that the authors have not been able to avail themselves of radiocarbon dating. Instead they have cited radiocarbon dates for crocodiles in the museum collection published by Richardin et al (2017). These dates correspond to the end of the Late Period to the middle of the Roman Period.

Claims made by the authors are fully supported by data and analyses through the open access tomographic database of the ESRF (http://paleo.esrf.eu), informative tables and excellent quality images included in the manuscript.

The methodology is sufficiently described to allow the experiment to be reproduced. The authors' experimental treatment of the Caiman crocodile confirms their interpretation of the mummification process followed by the ancient embalmers of the Nile crocodile. The synchrotron methodology is described in detail, although not every researcher has access to a synchrotron!

The manuscript is clearly written and organised in the traditional way, with abstract, introduction, method, results. However, we suggest the following minor changes and corrections that will enhance the text, as the authors are not native English speakers.

Publication is highly recommended after the following minor corrections have been carried out.

l. 42 Delete ‘content’, substitute ‘**composition**’.
l. 62 Delete **space** after ‘information’ and before the full stop.
l. 66 insert ‘**Lortet and Gaillard (17.)**’ Just the reference number is not sufficient.
l. 67 ‘However, as all the textiles have been removed, it is now impossible to reliably derive the origin …. through manufacturing techniques’. It would not have been possible to ‘reliably derive the origin’ unless there was evidence of an especially unusual and datable weaving or spinning technique. I would suggest: ‘**it is now impossible to gain insights into the origin of this mummy through any distinctive manufacturing techniques’**.
l. 68 Delete ‘elucidate on’, insert ‘**reveal**’.
l. 77 Delete ‘relative to’, substitute ‘**in**’
l. 77 Delete ‘hence we propose hereafter …’ insert (new sentence) ‘**Hence we propose the following updated description**’.
l. 78 Delete ‘already’, insert ‘**have**’.
l. 81 ‘plants’. Delete ‘**s’**, = singular **‘plant** shoots’.
l. 82 Delete ‘stick’.
l. 83 Delete ‘brake’, insert ‘**break**’. Misspelling.
l. 84 Delete **‘**indicating**’,** insert ‘**indicate**’. Delete ‘corps’, insert ‘**corpse**’, or better still, ‘**body**’.
L. 85 Delete ‘into’, insert ‘**in**’ the balm layer’.
l. 169 Insert **space** after ‘surface’ and before ‘Figure 1’.
l. 181 ‘Its dorsal aspects is stick’. **Delete s** = singular ‘dorsal aspect. Delete ‘is stick’, insert ‘adheres’, i.e. ‘**Its dorsal aspect adheres’**.
ll. 196-211 **NBB**

The composition of the ‘balm’ has not undergone chemical analysis. This should be acknowledged in the manuscript, as only the mineral inclusions that constitute natron have been identified (ll. 196-211, and Table 2.)
l. 238 Delete ‘maintained., substitute ‘**secured**’.
l. 255 ‘posture given to the body’ Suggest ‘**position in which the body was arranged**’.
l. 265 Delete ‘is’, insert ‘**are**’ (plural)
l. 352 Delete ‘Univeresety’, insert ‘**University**’. Misspelling.
l. 359 Ibid. ‘**University’**
